# Cancer in a drop: Liquid biopsy highlights from the American Society of Clinical Oncology (ASCO) 2025 annual congress

**DOI:** 10.1016/j.jlb.2025.100320

**Published:** 2025-08-06

**Authors:** Canio Martinelli, Roberto Borea, Maheswaran Shivahamy, Angelo Dipasquale, Erick F. Saldanha, Nadia Ghazali, Eleonora Nicolo, Pavel Stejskal, Letizia Pontolillo, Carolina Reduzzi

**Affiliations:** a International Society of Liquid Biopsy (ISLB) Young Committee, Spain; b Sbarro Institute for Cancer Research and Molecular Medicine and Center of Biotechnology, College of Science and Technology, Temple University, 1900 N 12th St, Philadelphia, PA, 19122, USA; c Department of Human Pathology of Adult and Childhood “Gaetano Barresi”, Unit of Obstetrics and Gynecology, University of Messina, Via Consolare Valeria 1, 98124, Messina, Italy; d Department of Internal Medicine and Medical Sciences (DiMI), School of Medicine, University of Genova, Genova, Italy; e Division of Medical Oncology, Department of Internal Medicine, The Ohio State University Comprehensive Cancer Center, College of Medicine, The Ohio State University Wexner Medical Center, Columbus, OH, USA; f Department of Medicine, University of Massachusetts Chan Medical School, Worcester, MA, USA; g Medical Oncology Unit, IRCCS Humanitas Research Hospital, Via Alessandro Manzoni 56, Rozzano, Milan, Italy; h Division of Medical Oncology and Hematology, Princess Margaret Cancer Centre, University Health Network, Toronto, CA, USA; i Department of Medicine, University of Toronto, Toronto, CA, USA; j Harvard T. H. Chan School of Public Health, Harvard University, Boston, USA; k Department of Medical Oncology, Princess Margaret Cancer Centre, Toronto, Canada; l Liquid Biopsy Platform, Department of Medicine, Weill Cornell Medicine, Englander Institute for Precision Medicine, New York Presbyterian Hospital, New York, NY, 10021, USA; m Institute of Molecular and Translational Medicine, Faculty of Medicine and Dentistry, Palacky University in Olomouc, 779 00, Olomouc, Czech Republic; n Laboratory of Experimental Medicine, University Hospital in Olomouc, 779 00, Olomouc, Czech Republic; o Department of Translational Medicine and Surgery, Università Cattolica del Sacro Cuore, Largo Francesco Vito, Rome, 00168, Italy

## Abstract

Over the past decade, liquid biopsy has progressively expanded its role in oncology, supported by mounting evidence demonstrating an increasing number of clinical applications. At the 2025 American Society of Clinical Oncology (ASCO) Annual Meeting, liquid biopsy emerged as a central theme across multiple sessions, with more than 700 abstracts, investigating the clinical utility of liquid biopsy across a wide range of tumor types and disease stages. Applications presented included cancer screening, minimal residual disease (MRD) detection, management of metastatic disease, and potential use for matching patients to clinical trials. This editorial, authored on the behalf of the Young Committee of the International Society of Liquid Biopsy (ISLB) highlights the result of selected studies, grouped by tumor type.

## 1 Lung cancer

In the phase 3 AEGEAN trial, perioperative durvalumab + chemotherapy demonstrated significant improvements in pathological complete response (pCR) (17 % vs 4.3 %) and event-free survival (EFS) (hazard ratio (HR) 0.68; 95 % confidence interval (CI) 0.53–0.88) in resectable non-small cell lung cancer (NSCLC) [1,2]. Using a tissue-informed assay for ctDNA MRD (minimal residual disease), the BEP (biomarker evaluable population) was only 21 % of patients, limiting interpretation due to the small sample size a concerning issue observed in all other studies employing tissue-informed MRD assays. Importantly, in the durvalumab arm, no patients who were MRD + at the post-surgical timepoint had a pCR or MPR (major pathologic response). Patients who were MRD-fared well in both arms of the study, while patients who were MRD + died early in both arms. Nevertheless, detectable ctDNA at the post-surgical timepoint was associated with more advanced disease stage at diagnosis, shorter disease-free survival (DFS) at 12 months, and mutations in the *KMT2* and *KEAP1* genes [3]. Unfortunately, these results do not support use of this methodology for therapeutic decision-making at the MRD timepoint. It is important to note that these exploratory ctDNA analyses were independent of the trial's primary endpoints (pCR and EFS). Similar issues have been observed in other studies using current tissue-based methodology (see Table 1).

**Table 1:** Summary of the Pivotal ASCO 2025 liquid-biopsy presentations for solid tumors.

Tumor type	Trial (phase)	Clinical setting	ctDNA/cfDNA platform	Key actionable finding	Ref
NSCLC	**AEGEAN** (III)	Perioperative immunotherapy (neoadjuvant chemo + durvalumab) vs chemo + placebo, in resectable stage II–III NSCLC.	Tumor-informed MRD assay (plasma ctDNA variants personalized via tumor WES); serial ctDNA sampling pre- and post-surgery.	*KEAP1* and *KMT2C* mutations were enriched in MRD-positive tumors and associated with poor benefit from immunotherapy, identifying a high-risk subgroup with poor prognosis despite treatment.	[2]
NSCLC	**CheckMate 77T** (III)	Perioperative immunotherapy (chemo + nivolumab) vs chemo + placebo, in resectable stage II–III NSCLC.	Tumor-informed MRD assay (plasma ctDNA variants personalized via tumor WES); serial ctDNA sampling pre- and post-surgery.	Dual ctDNA clearance + pCR conferred greatest EFS; ≥98 % of pts with residual ctDNA before surgery failed to reach pCR	[5]
NSCLC PD-L1+ (TPS ≥1 %)	Plasma-guided pembrolizumab- **NCT04166487**	Stage IV, treatment-naïve: pembrolizumab alone for two cycles → ctDNA read-out; non-responders escalate to pembro + platinum doublet chemotherapy	Tumor-agnostic 36-gene cfDNA panel (InVision™); molecular response = ≥ 50 % drop in max-VAF by cycle 2	ctDNA responders achieved 81 % ORR vs 21 % in non-responders; responders' median PFS 16.4 mo vs 4.8 mo non-responders; ∼20 % of pts required chemo-IO escalation, illustrating feasibility of ctDNA-triggered intensification	[8]
Colon	**Alliance N0147** (III)	Stage III CRC adjuvant setting (FOLFOX ± cetuximab)	Tissue-free methylation-based MRD test (Guardant Reveal™). 739 gene panel by NGS for ctDNA + samples (Guardant 360®)	Positive post-surgery ctDNA predicted of r recurrence and death; higher epigenomic TF reated with worsened prognosis	[9]
Colon	**DYNAMIC** (II**/**II)	stage III CRC adjuvant setting ctDNA-guided escalation approach vs standard management)	Tumor-informed MRD assay **(Safe-SeqS targeted CRC panel)**	ctDNA detection after surgery correlated to high 3-year RFS with no benefit from ctDNA guided escalation chemotherapy adjuvant treatment. Recurrence risk increased with rising ctDNA burden.	[10]
Colon (mCRC)	**PARERE** (II)	Stage IV *RAS/BRAF* ctDNA WT - panitutumab (*anti*-EGFR) followed by regorafenib vs. the reverse sequence (arm B).	Oncomine Colon cf-dDNA assay	In *RAS/BRAF* ctDNA-WT pts, panitumumab rechallenge improved PFS over regorafenib. 38 % of pts *RAS/BRAF* ctDNA mut.	[11]
Breast (early)	**I-SPY2** (II) “nodal-burden” analysis.	Neoadjuvant multi-arm therapy (stage II–III high-risk BC).	Tumor-informed Signatera® MRD panel (≤16 patient-specific variants; draws at BL, 3 wk, 12 wk, post-NAC)	Post-NAC ctDNA- negativity predicted lower residual nodal disease (ypN1-2) Findings support axillary surgery de-escalation (SLNB/TAD) in ctDNA- pts	[12]
Breast (early)	**PREDICT-DNA (TBCRC 040)**	Post-neoadjuvant HER2+ & TNBC (stage II–III)	Tumor-informed NeXT Personal® ultra-sensitive panel (≤1–3 ppm LoD; serial draws BL, post-NAT, post-surgery)	Post-therapy ctDNA detection (NPV 60 % for pCR) was a stronger relapse predictor than pCR itself	[14]
Breast (early)	**DARE**	Adjuvant ET surveillance in HR+/HER2- stage II–III BC. ctDNA-positive pts were randomized to continue standard ET vs switch to fulvestrant + palbociclib with negative imaging.	Tumor-informed Signatera® panel (≤16 patient-specific variants; serial draws during adjuvant ET)	ctDNA-guided switch doubled molecular clearance. Overall, ctDNA-negative pts showed 99 % 1-yr RFS, confirming high NPV.	[15]
Breast (metastatic)	**SERENA-6** (III)	1L HR+/HER2--mBC on AI + CDK4/6 inhibitor: pts randomized to switch to camizestrant vs continue AI, with ongoing CDK4/6 inhibitor at the moment of *ESR1* mutation detection in absence of radiological progression.	Guardant 360 NGS panel (plasma collection q2-3 months during surveillance phase)	*ESR1* ctDNA detecton - triggered switch to camizestrant before radiological disease progression doubled median PFS (16.0 vs 9.2 months)	[16, 17]
Breast (metastatic)	**DESTINY-Breast06** (III)	Exploratory biomarker analysis HR+, HER2-low/ultralow mBC: trastuzumab deruxtecan (T-DXd) vs physician's-choice chemotherapy	Baseline ctDNA profiling with GuardantOMNI™ 500-gene liquid-biopsy panel (tissue-free, comprehensive NGS)	T-DXd improved PFS and ORR over chemo regardless of *PI3K*, *ESR1*, or *BRCA1/2* status; largest benefit in BRCA-mutated tumors	[18]
Bladder	**NIAGARA** (III)	Peri-operative durvalumab + gem-cis vs gem-cis alone in cisplatin-eligible MIBC	Tumor-informed Signatera® MRD panel (≤16 patient-specific variants; draws at baseline, post-NAC, post-RC)	Baseline ctDNA-negative and post-NAC clearance predicted superior DFS; durvalumab increased clearance (70 % vs 57 %)	[22]
Cervical	**CALLA** (III)	Concurrent & adjuvant CRT ± durvalumab (stage IB2–IVA LACC)	Tumor-informed NeXT Personal® MRD panel; plasma at BL, C3 D1, C6 D1	Persistent ctDNA after CRT independently predicted progression; Durvalumab increased molecular clearance, especially in PD-L1 TAP ≥20 % tumors (ctDNA+ 19 % vs 41 % at C6 D1).	[23]
Endometrial	**DUO-E** (III)	1L carboplatin/paclitaxel + durvalumab → maintenance durvalumab ± olaparib	Methylation-based Guardant Infinity™ assay	Maintenance olaparib associated with additional ctDNA clearance, particularly in MMR-proficient disease. baseline ctDNA positivity correlated with shorter PFS.	[24]
Endometrial (screening)	Pap smear-derived cfDNA study	High-risk/diagnostic sampling before surgery	Pap-smear cfDNA panel (detecting tumor mutations → ctDNA)	Pap-derived ctDNA detected in >90 % of cases (81 % early stage) vs ∼25 % in plasma; Pap samples contained ∼5 × higher cfDNA yield, supporting a minimally invasive screening approach.	[25]
Sarcoma	Detecting ctDNA using personalized structural variants	High-risk, localized soft-tissue sarcoma after surgery ± neoadj RT (MRD window ≤8 wks post-op)	Tumor-informed SV panel (median 14 patient-specific breakpoints; WGS → multiplex dPCR)	Baseline ctDNA detected in 97 % (31/32) of pts. Within the MRD window, 100 % of ctDNA-positive pts relapsed vs 17 % if ctDNA-negative; median RFS 153 d vs 521 d, confirming high sensitivity and strong prognostic value for relapse forecasting.	[28]
Pan-cancer	MONSTAR-SCREEN-3 project (UMIN000053975)	Definitive cohort/MRD setting	Tumor-informed Ultra-sensitive bespoke WGS MRD assay (Precise™ MRD)	Revealed 100 % baseline sensitivity. 60 % of the ctDNA detection was at ultra-sensitive levels (<100 ppm)	[26]

Abbreviations: cfDNA = cell-free DNA; CRC = colorectal cancer; CRT = chemoradiation; DFS = disease-free survival; ESR1 = Estrogen Receptor 1; ET = endocrine therapy; HR/HER2 = hormone receptor/human epidermal growth factor receptor 2; LAAC = locally advanced cervical cancer; mBC = metastatic breast cancer; MMR = mismatch repair; MSI = microsatellite instability; MRD = minimal residual disease; NAC = neoadjuvant chemotherapy; NGS = next generation sequencing; NPV = negative predictive value; NSCLC = non-small-cell lung cancer; pCR = pathological complete response; PFS = progression-free survival; RFS = recurrence-free survival; TAP = tumor area positivity; TF = Tumor Fraction; WT = wild type.

The phase 3 CheckMate 77T trial evaluating perioperative nivolumab 66 % of nivolumab-treated patients achieved ctDNA clearance at surgery; however, only 50 % reached a pCR. In the placebo arm, 38 % cleared ctDNA, but only 12 % achieved pCR. Notably, over 98 % of patients with persistent pre-surgical ctDNA did not reach pCR. Importantly, the combination of ctDNA clearance and pCR was associated with the highest EFS [4,5]. In unresectable stage III NSCLC, early ctDNA detection during consolidative durvalumab after chemoradiotherapy (CRT) was significantly linked to shorter progression-free survival (PFS) (*p* = 0.004) and increased risk of death within 24 months (*p* = 0.017) [6].

In metastatic PD-L1+ (TPS ≥1 %) NSCLC, a pilot study of plasma-guided biomarker switch therapy demonstrated that the addition of chemotherapy to initial single agent pembrolizumab when ctDNA failed to fall by 50 % was associated with improved radiographic response in those with plasma ctDNA response (81 % vs 21 %) and also with increased PFS and OS, demonstrating the known prognostic effects of this ctDNA reduction. The small sample size limited interpretation of the chemotherapy addition. Overall, this study, highlighted the feasibility of a precision medicine approach to reducing chemotherapy exposure through plasma-guided strategies. Randomized studies of this strategy are warranted [7,8].

## 2 Colorectal cancer (CRC)

Two analyses underscored the prognostic significance of residual ctDNA for CRC in the adjuvant setting. In the Alliance N0147 trial**,** post-surgery ctDNA detection in stage III colon cancer patients receiving adjuvant FOLFOX ± cetuximab was significantly associated with worse DFS (HR 3.74, *p* < 0.0001), and poorer overall survival (OS) (HR 3.17, *p* < 0.0001). Moreover, stratification by ctDNA epigenomic tumor fraction refined the prognostic assessment, while mutations in *FLT1* and *PREX2* correlated with recurrence [9]. Similarly, the DYNAMIC III trial showed that ctDNA positivity after surgery correlated with worse recurrence free survival (RFS) (3-year RFS ≈50 %). However, no benefit from a ctDNA guided escalation treatment (oxaliplatin doublet or FOLFOXIRI) was reported (2-year RFS 52 % vs 61 %) [10]. In the metastatic setting, the PARERE trial, showed that liquid biopsy enabled the detection of *RAS* and/or *BRAF* V600E ctDNA mutations in over one-third of metastatic CRC patients otherwise considered eligible for *anti*-*EGFR* re-treatment. Moreover, in patients with wild type *RAS/BRAF* ctDNA, Panitumumab retreatment was associated with longer PFS compared to regorafenib, independently from the treatment sequencing [11].

## 3 Breast cancer

In early-stage breast cancer (eBC), the I-SPY2 trial assessed the association between ctDNA and axillary nodal burden, highlighting ctDNA as a non-invasive predictor of nodal status that could inform axillary surgical decision-making and potentially reduce overtreatment, although prospective validation is necessary [12]. An exploratory analysis in the hormone receptor-positive, HER2-negative (HR+/HER2-) cohorts from the same trial demonstrated ctDNA's potential utility for real-time monitoring of molecular responses to neoadjuvant endocrine therapy [13]. The PREDICT-DNA trial found ctDNA negativity post-neoadjuvant therapy to be a suboptimal predictor of pCR (negative predictive value: 60 %); however, ctDNA positivity strongly indicated higher recurrence risk compared to pCR status. In the DARE trial, ctDNA + high-risk HR+/HER2-eBC patients without radiographic disease showed higher ctDNA clearance rates and early correlation with clinical outcomes when treated with fulvestrant plus palbociclib in a ctDNA-guided escalation strategy, highlighting the potential of ctDNA to inform adjuvant treatment decisions [14,15]. In metastatic breast cancer (mBC), the phase-3 SERENA-6 trial provided the first global evidence for a ctDNA-guided approach to detect endocrine resistance and guide therapy adjustments prior to radiological progression in HR+/HER2-disease. An early switch to camizestrant rather than aromatase inhibitor continuation, based on *ESR1* mutation detection in ctDNA, significantly improved median PFS (16.0 months vs. 9.2 months; HR 0.44, 95 % CI 0.31–0.60, *p* < 0.00001) and delayed quality-of-life deterioration [16,17]. The DESTINY-Breast06 trial exploratory biomarker analysis demonstrated trastuzumab deruxtecan efficacy independent of baseline genomic alterations, with hints of greater benefit in *BRCA*-mutated tumors, highlighting the importance of comprehensive ctDNA profiling for tailored treatments [18]. Additional real-world and prospective studies linked ctDNA dynamics and clearance of *PIK3CA* mutations with improved therapeutic outcomes [20,21]. Findings from SWOG S1416 further highlighted ctDNA's capability to detect somatic homologous recombination deficiency (HRD)-related alterations, such as *CHEK2* and *BRCA1* mutations, in *BRCA*-wildtype triple-negative breast cancer, revealing patterns of mutual exclusivity and temporal evolution [19].

## 4 Genito-urinary and gynecologic cancers

In the phase 3 NIAGARA trial investigating perioperative durvalumab in muscle-invasive bladder cancer, ctDNA clearance post-neoadjuvant therapy (HR 0.32) and post-cystectomy (HR 0.09) correlated with improved survival outcomes. Durvalumab led to higher ctDNA clearance rates than control (70 % vs 57 %) and improved EFS regardless of baseline ctDNA status [22]. In the phase 3 CALLA trial for locally advanced cervical cancer, low baseline ctDNA levels (below the median) were associated with improved PFS and OS, while ctDNA detection during/after CRT (±durvalumab) was associated with higher risk of progression. Post-CRT ctDNA detection was lowest in the durvalumab arm, especially in patients with PD-L1 tumor area positivity (TAP) ≥20 % (19 % vs. 41 %) [23]. The DUO-E study showed a PFS benefit with durvalumab plus chemotherapy, followed by maintenance durvalumab ± olaparib, in advanced endometrial cancer (EC). Baseline ctDNA positivity correlated with an increased risk of progression. Durvalumab was associated with rapid reductions in ctDNA levels and reduced ctDNA rebound, while maintenance olaparib led to further ctDNA clearance in patients with MMR-proficient tumors [24]. Because no validated screening test exists for EC, women with Lynch syndrome currently undergo annual endometrial biopsy. In a feasibility study, tumor-specific mutations were detected in Pap-derived cell free DNA (cfDNA) in 93 % of cases - including 92 % of early-stage cancers - versus only 33 % in matched plasma samples. The Pap specimen yielded ∼5-fold more cfDNA (median 91 ng vs 19.5 ng) and therefore a much stronger ctDNA signal, raising the prospect of a minimally invasive screening tool for average- and high-risk women [25].

## 5 From pan-cancer to rare cancers

Important liquid biopsy data on rare cancers were presented. The multicenter Japanese MONSTAR-SCREEN-3 trial enrolled 1100 pan-cancer patients undergoing curative-intent surgery using an ultra-sensitive tissue-informed whole genome sequencing (WGS)-based MRD assay (Precise™ MRD) [26]. Personalized tumor-informed panels were successfully designed from matched tumor-tissue sequencing data in 97.2 % (69/71) of patients. Serial plasma samples were subsequently analyzed using these personalized panels [26]. With 114 patients enrolled (median follow-up: 3.4 months), the median number of panel-eligible alterations per patient was 6089. The assay demonstrated 100 % baseline sensitivity (41/41), detecting tumor fractions ranging from <0.001 % to 45.2 %, with 24.4 % MRD positivity at 1-month post-surgery [26]. In early-stage high-risk soft-tissue sarcoma (STS), a Canadian retrospective study evaluated a tumor-informed structural variant (SV) ctDNA assay in 32 patients (228 plasma samples) with a median follow-up of 20.1 months [27]. ctDNA detection was 97 % at diagnosis (31/32), with MRD positivity (within 8 weeks postoperatively) in 18 % (4/22), all of whom developed metastatic recurrence (median lead time: 136 days; range 28–210 days). Among 18 MRD-negative patients, 3 later recurred with ctDNA detection preceding radiologic recurrence (median lead time: 87 days) [27]. In the NCI-MATCH trial evaluating ctDNA for clinical trial matching, 60 % (2286) of enrolled patients had rare cancers. Plasma ctDNA (TSO500 ctDNA v2) from 2194 patients was sequenced alongside tumor samples (Oncomine Comprehensive Assay v2) [28]. Analysis of five different tumor types showed only 1.4 % testing failure. Despite cfDNA shedding variations, most samples met the minimum cfDNA threshold (10 ng); highest shedding was noted in cholangiocarcinoma and small cell lung cancer. Positive percent agreement between ctDNA and tissue tests was 83.4 % (range 76.5–97.9 %), correlating with tumor fraction (median fraction: 6.49 % for concordance >75 %, vs. 0.37 % for concordance <75 %), underscoring ctDNA's potential for precision therapy trial enrolment.

## 6 ASCO 2025 key takeaways

**Fig. 1:**
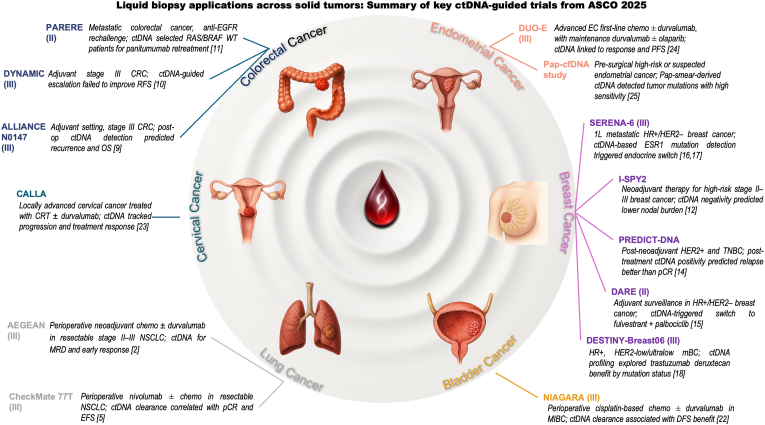
Liquid biopsy across solid tumors at ASCO 2025. Selected ctDNA-guided phase II and III trials are grouped by tumor type to illustrate minimal residual disease assessment, response monitoring, therapy adaptation, and trial matching. Numbers in brackets refers to references. ctDNA = circulating tumor DNA; cfDNA = cell-free DNA; MRD = minimal residual disease.

ASCO 2025 highlighted a significant increase in liquid biopsy applications across oncology (Fig. 1), pointing out promising practice-changing potential alongside clear limitations. Plasma-guided therapeutic strategies in NSCLC demonstrated the potential to personalize chemotherapy regimens effectively. In metastatic HR+/HER2- BC, timely ctDNA-based *ESR1* mutation monitoring and treatment of molecular progression rather than radiological progression substantially reduced disease progression risk. Additionally, ctDNA negativity might soon justify less invasive axillary surgery approaches in early-stage BC. Postoperative ctDNA emerged as a robust prognostic marker in stage III colon and muscle-invasive bladder cancers. Although preliminary, these studies represent a significant step forward in integrating liquid biopsy into clinical practice. Despite the promising data, several limitations persist, including assay heterogeneity, a limited number of ctDNA-positive samples, and inconsistencies between ctDNA clearance and pathological response, emphasizing the biological complexity and the need for cautious interpretation. Standardization efforts, such as harmonizing thresholds and sampling time points, are essential to enable broader clinical adoption. Prospective trials incorporating ctDNA-driven decision-making will be key to establishing the clinical utility of liquid biopsy. In parallel, emerging multi-omic platforms that integrate methylation profiling, fragmentomics, and immune-related signatures hold promise for more precise risk stratification and individualized treatment approaches. Additionally, novel tools like Pap smear-derived cfDNA offer exciting potential for population-scale cancer screening.

## Ethical approval

No ethical approvals or patient consent were necessary for the study.

## Declaration of competing interest

The authors declare the following financial interests/personal relationships which may be considered as potential competing interests: The authors declare the following financial interests/personal relationships which may be considered as potential competing interests: Carolina Reduzzi reports a relationship with Menarini Silicon Biosystems Inc that includes: funding grants. Carolina Reduzzi reports a relationship with ANGLE plc that includes: non-financial support. Carolina Reduzzi reports a relationship with Tethis spa that includes: nonfinancial support. Letizia Pontolillo reports travel support from Pfizer, Eli Lilly, and Gilead, and speaker fees from Daiichi Sankyo and Novartis. All the other authors declare that they have no known competing financial interests or personal relationships that could have appeared to influence the work reported in this paper. If there are other authors, they declare that they have no known competing financial interests or personal relationships that could have appeared to influence the work reported in this paper.
